# Effects of Danlou tablet for the treatment of stable angina pectoris: A study protocol of a randomized, double-blind, and placebo-controlled clinical trial

**DOI:** 10.1097/MD.0000000000023416

**Published:** 2020-12-04

**Authors:** Guang Yang, Haoqiang He, Hongzheng Li, Zinuo Shen, Siyuan Zhou, Bingxu Lu, Jun Li, Qingyong He, Zhenpeng Zhang, Yongmei Liu, Jie Wang, Hengwen Chen

**Affiliations:** a Department of Cardiology, Guang’anmen Hospital, China Academy of Chinese Medical Sciences; b Graduate School, Beijing University of Chinese Medicine, Beijing, China.

**Keywords:** Danlou tablet, protocol, stable angina, traditional Chinese medicine

## Abstract

**Introduction:**

Stable angina pectoris has a high prevalence and causes serious harm. Revascularization therapy can relieve angina pectoris to some extent, but it is not widely accepted in China due to the cost and secondary events. The Chinese proprietary medicine Danlou tablet has been widely used to treat angina pectoris, but previous trials had inadequate methodologies. In this study, we aim to conduct a randomized controlled trial to evaluate its efficacy and safety on stable angina.

**Methods and analysis:**

This study is a WeChat-based randomized, double-blind, and placebo-controlled clinical trial in China. Eligible participants are adults (aged 30–75 years) with CT-confirmed stable angina and traditional Chinese medicine-diagnosed intermingled phlegm and blood stasis syndrome. A total of 76 participants will be randomly allocated in a 1:1 ratio to the oral Danlou tablet group (1.5 mg a time, 3 times daily for 28 days) or the placebo group. Patients are permitted concomitant use of routine medications during these 28 days. The primary outcome is angina frequency per week. The secondary outcomes include angina severity, angina duration, traditional Chinese medicine efficacy, the withdrawal rate of emergency medications, blood lipids, and electrocardiograph efficacy. The WeChat app will be used to remind patients to take their medicines and fill out the forms. All data will be recorded in case report forms and analyzed by Statistical Analysis System software.

**Ethics and dissemination:**

This study has been approved by the Ethics Committee of Guang’anmen Hospital, China Academy of Chinese Medical Sciences in Beijing, China (No. 2019-225-KY).

**Trial registration number:**

ClinicalTrials.gov, ID: ChiCTR1900028068.

## 1 Introduction

Coronary arterial disease (CAD) is the most common chronic noncommunicable disease, also the leading cause of death worldwide. The Global Burden of Disease Study 2017 reported that CAD had affected more than 110 million people and caused about 8.92 million deaths by 2016.^[1]^ Stable angina pectoris (SAP) is one of the main types of CAD. Angina is also the most painful symptom for the patients. In 2016, 9.4 million American adults and 11 million Chinese adults suffered from angina.^[2,3]^ Some surveys have shown that SAP associated with increased risks of cardiovascular endpoint events and all-cause mortality.^[4,5]^ That means we need to pay more attention to managing this disease. The guideline recommends lifestyle interventions, intensive medications, and reduction of risk factors as the basic treatments.^[6]^ Some patients still have persistent angina even after the basic treatments. Although percutaneous coronary intervention has proven to relieve angina and reduce the extent of myocardial ischemia,^[7]^ it is not widely accepted by patients in China. In 2017, an average of only 684 people per million cardiovascular patients had stent implantation.^[3]^ This could partly be due to the high cost and risk of revascularization: people may face a 5% to 10% risk of restenosis within 1 to 5 years^[8,9]^ and still have substantial mortality and morbidity rates.^[10,11]^ These problems suggest that there is an unmet need to develop new therapies for SAP.

In China, a proprietary medicine named Danlou tablet (DLT), has long been considered as a common therapy for SAP. DLT is composed of 10 kinds of Chinese herbal medicines (see Table 1), and the indication for it is treating coronary artery disease with “intermingled phlegm and blood stasis” syndrome. Clinical studies have shown DLT could reduce the frequency and duration of angina pectoris, and improve cardiac function. In recent years, several studies on DLT have been carried out.^[12,13]^ Animal experiments have shown that DLT is efficient in strengthening the protection of vascular endothelium, reducing the levels of inflammatory mediators, and stabilizing atherosclerotic plaques.^[14,15]^ A metabolomics study has revealed that the mechanisms underlying the effects of DLT in the treatment of CAD involve the inhibition of epidermal growth factor receptor expression and the activation of the mitogen-activated protein kinase signaling pathway by regulating glycerophospholipid metabolism and energy metabolism.^[16]^

**Table 1:** Components of Danlou tablet.

Chinese name	English name	Origin	Pharmacological effects
Gua Lou Pi	*Trichosantheskirilowii*	The dried peel of *Trichosantheskirilowii Maxim*	Antiatherosclerotic, and vascular endothelium protection^[17]^
Xie Bai	*Allium macrostemon*	The dried bulb of *Allium macrostemon*	Antiatherosclerotic, antioxidant, antihyperlipidemic, and anticoagulation^[18]^
Ge Gen	*Pueraria montana var. lobata*	The dried root of *Pueraria montana var. lobata*	Antihypertensive, cardiac protection, and antiatherosclerotic^[19]^
ChuanXiong	*Conioselinumanthriscoides* “*Chuanxiong*”	The dried rhizome of *Conioselinumanthriscoides* “*Chuanxiong*”	Antiplatelet aggregation, antithrombotic and anti-inflammatory^[20]^
Dan Shen	*Salvia miltiorrhiza*	The dried root or rhizome of *Salvia miltiorrhiza*	Antiplatelet aggregation, myocardium protection and antiatherosclerotic^[21]^
Chi Shao	*Paeonia anomala subsp. veitchii*	The dried root of *Paeonia lactiflora*	Promoting angiogenesis, analgesia and anti-inflammatory^[20]^
Ze Xie	*Alisma plantago-aquatica subsp. orientale*	The dried tuber of *Alisma plantago-aquatica subsp. orientale*	Antiatherosclerotic, antihypertensive, and anti-inflammatory^[22]^
Huang Qi	*Astragalus mongholicus*	The dried root of *Astragalus mongholicus*	Myocardium protection, promotion of microcirculatory and anti-inflammatory^[23]^
Gu Sui Bu	*Drynariaroosii*	The dried rhizome of *Drynariaroosii*	Promoting angiogenesis and anti-inflammatory^[24]^
Yu Jin	*Curcuma aromatica*	The dried root tuber of *Curcuma aromatica*	Analgesia, promoting blood circulation and lower blood viscosity^[25]^

Although several studies have reported the effective role of DLT in the treatment of stable angina, the evidence is not sufficiently strong to support clinical decision making for the low quality of methodology. WeChat is an instant messaging app used by practically everyone in China, capable of transmitting various texts, voices, pictures, and video material. Using WeChat, we will conduct an objective superiority trial to investigate the efficacy and safety of DLT in patients with SAP.

## 2 Methods

### 2.1 Study design

**Figure 1:**
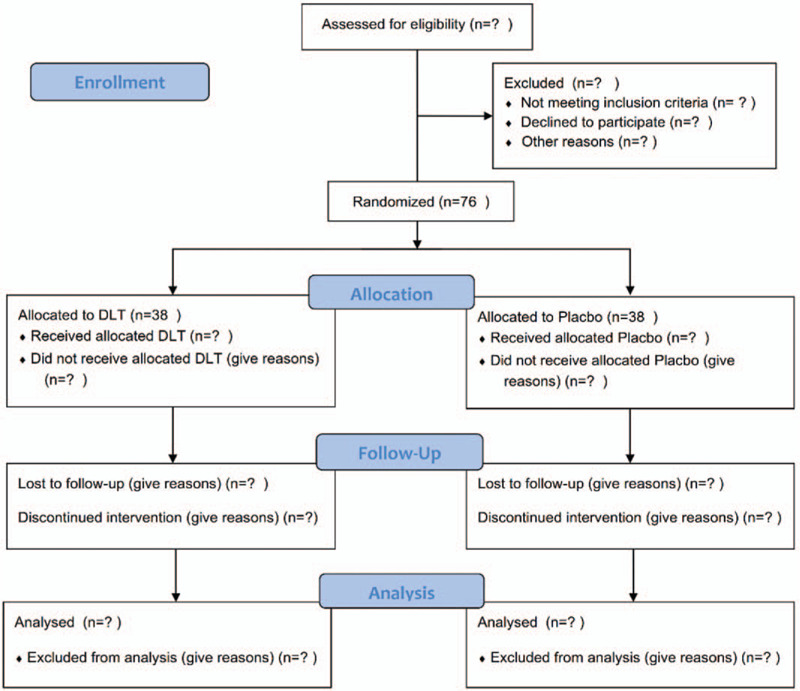
flow diagram for DLT study design. DLT = Danlou tablet.

Through a randomized, double-blind, and placebo-controlled clinical trial, we will investigate the curative efficacy and safety of DLT in patients with SAP. The trial will include a 1-week screening period and a 4-week treatment period. We plan to enroll 76 participants, who will be allocated randomly in a 1:1 ratio by a central randomization system (CRS) into the intervention group and the control group. Following the original medication regimen, participants will take DLT or placebo 3 times per day. This trial has already been registered at the Chinese Clinical Trial Registry on December 9, 2019 (ChiCTR-1900028068), following the principles of the Declaration of Helsinki and Good Clinical Practice guidelines. We will strictly follow the Consolidated Standards of Reporting Trials (CONSORT 2017)^[26]^ and the Standard Protocol Items: Recommendations for Interventional Trials (SPIRIT 2013)^[27]^ for the research design. A flow diagram illustrating our study design is presented in Figure 1.

### 2.2 Participant recruitment

In the present study, 76 participants will be recruited between September 2020 and December 2021 in Guang’anmen Hospital, China Academy of Chinese Medical Science, Beijing. The main forms of recruitment will be advertisements posted on WeChat, webpages, and notice boards. The advertisements will focus on a brief description of what this study aims for and how to participate. In addition, cardiologists in the hospital will identify outpatients with SAP and suggest them to participate in the study. After 2 researchers both confirm that a participant meets the inclusion and exclusion criteria, he or she can sign the informed consent form and join the trial.

### 2.3 Inclusion criteria

The inclusion criteria are the following:

(1) patients diagnosed with SAP with “intermingled phlegm and blood stasis syndrome;”
(2) age ranging from 30 to 75 years;
(3) voluntary participation;
(4) using WeChat.

In respect of the diagnosis of SAP, we refer to the 2019 European Society of Cardiology Guidelines for the Diagnosis and Management of Chronic Coronary Syndromes,^[6]^ the Society for Cardiovascular Angiography and Interventions/American College of Cardiology/American Heart Association Expert Consensus Document: 2014 Update on Percutaneous Coronary Intervention Without On-Site Surgical Backup.^[28]^ Participants with a pre-test probability of >15% are recommended to undergo a coronary dual-source CT angiography. Based on the CT angiography results, patients are diagnosed with SAP in the case of at least 1 major coronary branch stenosis of more than 50%. Participants should also have typical SAP symptoms.

In respect of the diagnosis of “intermingled phlegm and blood stasis syndrome,” we refer to the Diagnostic Criteria for Syndrome Elements of Coronary Heart Disease Angina published by the Chinese Society of Traditional Chinese Medicine Cardiovascular Disease Branch in 2018.^[29]^ Syndrome elements are the smallest diagnostic units for the diagnosis of traditional Chinese medicine (TCM) syndromes. A diagnosis of “phlegm syndrome” and “blood stasis syndrome” is made if the summed scores of single syndrome elements ≥8 points. When the criteria of both syndromes are met, the “intermingled phlegm and blood stasis syndrome” is diagnosed.

### 2.4 Exclusion criteria

Participants meeting any of the following criteria will be excluded from the trial.

(1) Patients who have previously experienced unstable angina pectoris and/or acute myocardial infarction; other diseases that may cause chest pain, such as congenital heart diseases, valvular diseases, and severe neurosis; arrhythmia; heart function of grade III or IV.
(2) Acute stage of cerebral infarction.
(3) Patients with severe liver- and/or kidney-related diseases; patients whose alanine aminotransferase and aspartate aminotransferase levels are higher than 1.5 times the upper limit of the normal range; patients with abnormal renal function.
(4) Acute infection within the last 2 weeks.
(5) Patients with other serious diseases that require treatment (like tumors, hematonosis, diabetes complications, etc).
(6) Mental disorders or mental retardation which prevent the participant from completing the questionnaire.
(7) Pregnant and lactating women.
(8) Tendency to bleed; abnormal disseminated intravascular coagulation or international normalized ratio; low platelet levels.
(9) Participation in other clinical trials within the last month.
(10) Allergic constitution or allergy to the constituents of the drugs.
(11) Unable to read or listen to the materials sent via WeChat (words, audio, and images).

### 2.5 Removal and discontinue criteria

A participant will be removed under the following circumstances:

(1) violations of the inclusion and/or exclusion criteria;
(2) refusal to undergo required examinations;
(3) failure to take drugs during the treatment period;
(4) myocardial infarction or sudden cardiac events.

A participant has the right to drop out at any time during the treatment; however, his or her reasons for withdrawal must be recorded in the case report form (CRF). According to the intention-to-treat principle, their last data will be involved in the final analysis. Under the following circumstances, the trial might be discontinued:

(1) participants begin to have serious adverse reactions after taking DLT;
(2) participants begin to show high sensitivity to the medication, such as headache, stomach ache, diarrhea, and other discomforts;
(3) masking and randomization fail.

### 2.6 Randomized design, blinding, and allocation concealment

In order to reduce selection bias and balance various non-research factors among groups, we will adopt a randomized block design and allocation concealment. The participants will be randomized in a 1:1 ratio into the DLT group and the placebo group, in 4 blocks of 19 participants. Random numbers from 1 to 76, as well as group numbers, will be generated by Statistical Analysis System software in CRS managed by the Clinical Evaluation Center of the China Academy of Chinese Medical Science. After inputting a participant's information into CRS, a random number will be generated. After the participant signs informed consent, the researcher obtains the corresponding group number from CRS. Neither of the participants and researchers will be allowed to know the random numbers and group numbers. In order to keep the study double-blind, DLT and the placebo will have the same appearance, smell, taste, and weight. The allocation will not be revealed until the end of the study, unless serious adverse events (AEs) happen. If an individual begins to show serious adverse reactions, the case will be reported to the Ethics Committee, and the blinding will be uncovered by the principal researcher. Then the participant will be removed from the study, and the related data will be recorded. If the emergency events exceed 20% of the sample content, the clinical trial will be discontinued, and the results will be deemed invalid.

### 2.7 Interventions

The trial will start with a 1-week screening period, during which other TCM medications for CAD are discontinued. Subsequently, all participants will take DLT or placebo for 4 weeks. Both groups continue routine medications, including long-acting nitrate drugs, lipid lowering drugs, and anti-platelet drugs. Cornell Chemical Company, who produces DLT and DLT placebo (both 0.3 g × 15 tablets, 5 tablets at a time, 3 times per day), guarantees that DLT (batch number, 20190905) and DLT placebo (batch number, 20190801S) have the same package, color, shape, and taste. DLT is manufactured according to the standards of the Pharmacopoeia of the People's Republic of China (version 2015). The placebo consists of starch, dextrin, and food coloring (mass ratio, 2:1:0.006). The drug counter will be locked and stored at room temperature under the management of a drug manager. In week 0 and week 2, researchers will hand out the drugs, according to the group numbers generated by CRS. All participants will be invited to a WeChat group, and a researcher will send messages every morning to remind patients to take the medicine. We will record and evaluate the administration of drugs in 2 ways.

**Figure 2:**
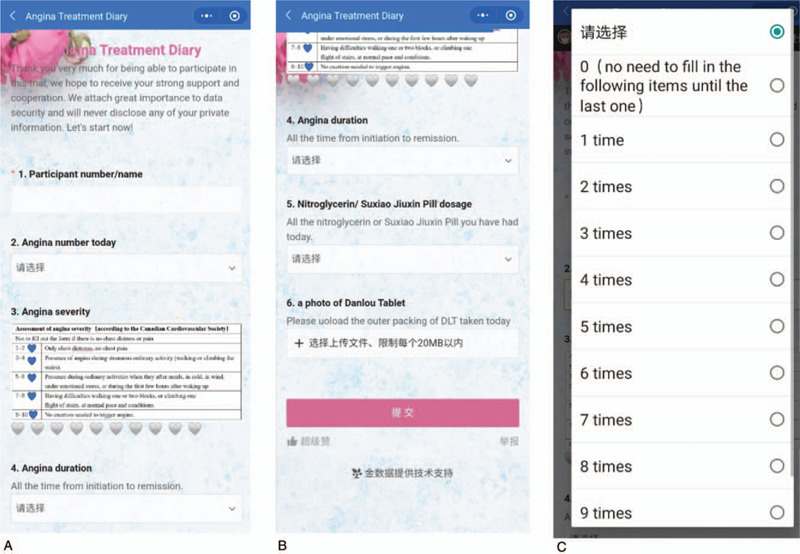
The interface of angina treatment diary in WeChat. (A, B) Main interface, (C) Angina number today.

(1) We will send an “angina treatment diary” in the form of a questionnaire (see Fig. 2) to the WeChat group every night, and the participants have to upload a picture of the medicines taken that day.
(2) In week 2 and week 4, the drugs that the participants have received, taken, and returned will be recorded in CRFs.

### 2.8 Data collection

#### 2.8.1 Primary and secondary outcomes

All participants will be required to record the frequency, severity, and duration of angina in the “angina treatment diary.” The “angina pectoris diary” will be presented in the form of an electronic questionnaire, which will be sent to the patients every night via WeChat. The primary outcome is the change in the frequency of angina attacks from baseline to week 4. Secondary outcomes include the severity and duration of angina attacks, TCM efficacy, the withdrawal rate of emergency medications, blood lipid efficacy, and ECG efficacy. The severity of angina will be reported on the basis of the Visual Analogue Scale and the Canadian Cardiovascular Society score (1–2 points, only chest distress, no chest pain; 3–4 points, presence of angina during strenuous ordinary activity; 5–6 points, presence of angina during ordinary activities after meals, in the cold, in the wind, under emotional stress, or during the first few hours after waking up; 7–8 points, having difficulties walking 1 or 2 blocks or climbing one flight of stairs, at normal pace and under normal conditions; 9 to 10 points, no exertion needed to trigger angina). Angina duration refers to the time between the initial onset of symptoms to the time of remission. TCM efficacy is reported on the basis of the Curative Effect of Traditional Chinese Medicine on Coronary Heart Disease Stable Angina issued in 2018.^[30]^ The withdrawal rate of emergency drugs is calculated as follows: withdrawal rate = (number of tablets before treatment − number of tablets after treatment)/(number of tablets before treatment) × 100%. Emergency drugs mainly include nitroglycerin and the SuxiaoJiuxin Pill (another common emergency medicine in China). Blood lipids refer to total cholesterol, triglyceride, low-density lipoprotein–cholesterol, and high-density lipoprotein–cholesterol levels. The ECG efficacy standards are based on the Evaluation Criteria for Efficacy of Coronary Heart Disease Angina Pectoris and ECG (see Table 2). Among these indicators, the angina-related outcomes are derived from the electronic “angina treatment diary,” and other indicators are derived from the CRF. The data collection schedule is shown in Table 3.

**Table 2:** ECG efficacy criteria.

Criteria	ECG changes
Marked effect	The ECG returns to “substantially normal” or reaches “normal ECG.”
Validation	The decrease of ST segment is to increase by more than 0.05 mV after treatment, but it does not reach the normal level. In the main electrocardiographic lead, the T wave changes lighter (up to 25%); or the T wave changes from flat to upright, improvement of atrioventricular or ventricular block.
Invalidation	The ECG is basically the same as before treatment.
Aggravation	The ST segment is to decrease by more than 0.05 mV after treatment. In the main electrocardiographic lead, the T wave changes deeper (up to 25%); or the T wave changes from upright to flat, or the upright T wave becomes inverted, and an ectopic rhythm, atrioventricular or ventricular block.

**Table 3:** Schedule of data collection.

	Screening stage		Inclusion day	Treatment period	
Items	-7th Day	-1th Day	0th Day	2th Week	4th Week
Informed consent	√				
Inclusion/exclusion criteria			√		
Demographic data	√				
General clinical data	√				
Anginal frequency			√	√	√
Anginal severity and duration			√	√	√
TCM effect			√	√	√
Electrocardiograph			√		√
Blood Lipid		√			√
Vital signs		√			√
Blood pressure		√			√
Blood and urine routine		√			√
Stool routine and occult blood		√			√
Liver and renal function		√			√
Record adverse events				√	√

Checked boxes indicate the indices tested during a specific time period. General clinical data include medical history, course of the disease, treatment history, concomitant diseases, and concomitant medications. Vital signs include body temperature, respiration rate, heart rate, and blood pressure. CRF = case report form, TCM = traditional Chinese medicine.

### 2.9 Safety outcomes

The safety outcomes include vital signs and laboratory examinations, all of which will be performed in weeks 0 and 4. Vital signs include body temperature, respiration rate, heart rate, and blood pressure. Laboratory tests—ECG and blood routine, urine routine, and stool routine tests—will be used to evaluate occult blood, liver function (alanine aminotransferase, aspartate aminotransferase, γ-glutamyl transpeptidase, total bilirubin, and alkaline phosphatase levels), and renal function (urea nitrogen and Crea levels). The severity and incidence of AEs or reactions will be recorded in detail.

### 2.10 Patient and public involvement

As a preliminary study, we surveyed 7 outpatients with angina pectoris and created the “angina treatment diary” on WeChat. This questionnaire has 6 items:

(1) participant number/name;
(2) angina number today;
(3) angina severity;
(4) angina duration;
(5) nitroglycerin/SuxiaoJiuxin Pill dosage;
(6) a photo of the DLT.

Because the drop-down option is set, a patient can complete it within 1 minute, so this questionnaire will not bore patients. In addition, we will investigate the TCM efficacy. Because participants may not grasp how to judge the changes of their “syndromes,” which will hinder the dissemination of research conclusions to some extent, we converted the abstract concept of “syndrome” into an assessment scale containing clinical symptoms and signs, which we present in a simple and clear way. This scale was created based on early clinical practice, cross-sectional surveys, and literature searches. It has also been examined and improved by patient representatives. The results of this TCM scale can make the effects of DLT understood by patients who want to try TCM therapy, which would promote the use of DLT. In brief, patient and public involvement are necessary for the design, conduct, report, and dissemination of this research. After the statistical analysis, the results will be disseminated to the public via academic conferences and peer-reviewed journals. The patients will be informed of the final results through WeChat.

### 2.11 Sample size estimation

As the trial is a superiority trial, we use the non-zero null test in Power Analysis and Sample Size (version 11) for sample size estimation. In a previous study,^[31]^ the mean (and standard deviation) of the angina frequency in the treatment group was 1.90 ± 0.45 per week, and that in the reference group was 2.55 ± 1.03 per week. We wanted to make the most conservative estimate, so we considered the superiority margin as 0 and the true difference as 0.65. Based on a 2-sided test size of a level of 5% (*α* = 0.05), a power of 90% (*β* = 0.1), and equal sample sizes in the 2 groups (*n*_1_:*n*_2_ = 1:1), our study will require 27 participants in each group. We adjusted the sample size of each group to 30. Considering a dropout rate of maximum 20%, the final sample size was estimated to be 76 participants.

### 2.12 Adverse events

AEs are defined as negative clinical manifestations following the treatment, and adverse reactions are defined as drug-related AEs. Participants can report AEs via WeChat at any time. We will record the occurrence time, severity, duration, measures taken, and outcomes of AEs in the CRF. AEs will be divided into mild, moderate, and severe. The causal relationship between AEs and drugs will be judged as affirmative, very likely, possible, suspicious, or impossible. Mild to moderate adverse reactions will be continuously tracked by the research team. Once judged as a serious adverse reaction, it will be reported to the Ethics Committee, and the participant's trial will be terminated immediately. When a participant begins to show an emergency, the principal investigator will read the participant's emergency letter, give appropriate treatment based on the drug taken and the symptoms that appear, and record the reason for unblinding.

### 2.13 Quality control of data

All researchers will receive training courses to remember the standard operation procedure. In addition, they will need to master the screening of eligible participants, the use of CRS, the distribution and recording of the drugs, data filling, and other matters. They should also fill in CRFs in accordance with uniform requirements. Once the CRFs are formed, they cannot be changed; any additional revisions should be described, signed, and dated in time. Any subsequent amendments, along with the accompanying material provided to the patient, will be submitted to the Research Ethics Committee. During the trial, an independent Data Monitoring Committee will be set up to check the following issues: whether the contents of CRFs are consistent with the original data; whether the participants meet the inclusion criteria; the signatures of the participants’ informed consent; and the distribution, storage, and recording of drugs. In addition, we will maintain the dropout rate below 20% by communication through WeChat.

### 2.14 Monitoring

The Data Monitoring Committee of the trial is composed of 3 independent monitors/auditors (HL, BL, QH). The Trial Steering Committee is composed of 6 experts in the fields of pharmacology (HC), molecular biology (YL), cardiology (JL), and interventional cardiology (ZZ), 4 researchers (GY, HH, ZS, SZ) involved in the clinical trial, 3 auditors, and 1 statistician (HC). This committee will have 2 meetings before patient enrollment and data analysis to examine the completeness and blindness of the trial. No interim analysis is planned. When a participant begins to show an emergency (eg, a severe adverse reaction or an allergic reaction), the principal investigator (JW) has the right to read the emergency letter, give appropriate treatment, and decide whether to terminate the patient's trial.

### 2.15 Data collection and management

In week 0, week 2, and week 4, all demographic data and outcome measures will be recorded in CRFs according to the description of patients, and the angina-relevant indices will be exported in an excel from the WeChat background and filled in the CRFs. Since patients have to fill in their “angina treatment diary” every day, the accuracy and reliability of angina-related indicators are guaranteed. The researchers will also send private messages to participants to remind them of their fillings, which will guarantee their participation. The researchers will check the photos of DLT taken daily to ensure patient compliance with the medication regimen. After the data monitor and auditors have checked the CRF contents, 2 independent students will enter data separately in EpiDate software (Version 3.1), and the statistician will check the consistency of the 2 pieces of entered data. The Trial Steering Committee will convene a meeting to evaluate the blindness and the completion of the trial, and then lock the database.

### 2.16 Statistical analysis

Statistical analyses consist of 3 parts—baseline comparability analysis, efficacy analysis, and security analysis. An independent statistician will be in charge of the analyses, and Statistical Analysis System (version 9.2) will be used. All tests are 2-sided, and *P* < .05 is considered to indicate statistical significance. According to the intention-to-treat principle,^[32]^ there will be 3 analysis sets: the full analysis set, the per-protocol analysis set, and the safety analysis set. Participants who insist in taking medication for 2 weeks will be involved in the full analysis set group; if a critical variable is missing in any given case, the statistician will consider the last observation result as the final data. Patients who conform to the treatment plan will be included in the per-protocol analysis set group. The Mantel–Haenszel Chi-squared test and analysis of covariance will be conducted in the case of important baseline non-comparability. Normally distributed quantitative data will be presented as mean ± standard deviation and analyzed by Student *t* test. Non-normally distributed quantitative data will be presented as median and interquartile range and analyzed by the Kruskal–Wallis test. Ordinal data, like ECG efficacy, will be analyzed by the Kruskal–Wallis test. Qualitative data will be presented as percentage and rate and analyzed by the Chi-square test and Fisher exact test. The paired Student *t* test will be used to analyze significant differences between pre- and post-treatment. Moreover, subgroup analysis will be conducted according to gender, age, and complications to explore the internal consistency of the conclusions. For the safety analysis, the statistician will adopt the safety analysis set to evaluate the incidence rate of AEs and the change of laboratory indices. Both AEs and laboratory indices are described in lists and analyzed by the chi-square test or Student *t* test.

## 3 Discussion

This study is a WeChat-based block-randomized, double-blind, placebo-controlled, parallel-group clinical trial with the aim of investigating the efficacy of DLT on stable angina. DLT is a Chinese proprietary medicine for the treatment of “intermingled phlegm and blood stasis syndrome” in CAD. Nitroglycerin is usually applied for the acute phase of angina attacks, while long-acting nitrates and trimetazidine are utilized for the relatively stable period. DLT is taken 3 times per day, just like the latter. DLT has been proven effective in clinical trials, animal experiments, cell experiments, pharmacology studies, a metabolomics study, and a network pharmacology study. However, previous clinical studies of SAP may have limitations with respect to randomization, allocation concealment, blinding, and so on. Moreover, previous studies took therapeutic effective rates calculated from ECG efficacy, angina pectoris scores, and TCM syndrome scores as the primary outcomes. Different evaluation standards and study designs lead to greater systematic error. Therefore, we will choose angina frequency as the primary outcome for this study, as it quantitatively, objectively, and specifically reflects the treatment efficacy. Moreover, we will evaluate the degree of coronary artery stenosis by dual-source CT angiography and exclude patients who are diagnosed with myocardial infarction or have a stent implantation, in order to reduce selection bias and confounding factors and to ensure compliance with the patients’ inclusion criteria.

Currently, the combination of clinical medicine and Big Data is getting closer and closer, and WeChat could be considered as a “transfer station” of Big Data. WeChat is a smartphone application for instant messaging and transferring of files. During the COVID-19 pandemic, WeChat has become an indispensable tool for tracking travel records of people and inferring the development of new trends.^[33]^ Several studies have used WeChat to track the level of self-management of patients with hypertension,^[34]^ diabetes,^[35]^ and chronic obstructive pulmonary disease.^[36]^ Although WeChat has powerful functions and promising potential in the clinical field, no clinical research has been reported where the patients’ daily angina and medication were recorded using a questionnaire in WeChat. When participants fill in forms via WeChat, this can avoid forgetfulness and reduce the nonresponse bias and the measurement bias. In addition, it can prevent researchers from recording errors and falsifying data, which ensures the validity of results.

The limitations of this study can be summarized as follows.

(1) The trial is solely conducted in 1 hospital in Beijing, so the conclusions may not be generalizable globally.
(2) This trial only observes the short-term effectiveness, the benefit of endpoint events should be further evaluated in the future studies.

## Acknowledgments

The authors appreciate that: China Food and Drug Administration (CFDA), who supported all the costs of the trial; Cornell Chemical Company of Jilin Province, who provides the drugs and the placebos; all authors of the references. We thank LetPub (www.letpub.com) for its linguistic assistance during the preparation of this manuscript.

## Author contributions

**Conceptualization:** Guang Yang, Haoqiang He, Zinuo Shen, Siyuan Zhou, Bingxu Lu, Jun Li, Qingyong He, Zhenpeng Zhang, Yongmei Liu, Jie Wang.

**Data curation:** Guang Yang.

**Formal analysis:** Guang Yang.

**Methodology:** Jie Wang, Hengwen Chen.

**Resources:** Jie Wang, Hengwen Chen.

**Supervision:** Zinuo Shen.

**Validation:** Guang Yang, Zinuo Shen, Jie Wang, Hengwen Chen.

**Visualization:** Guang Yang, Siyuan Zhou, Jie Wang.

**Writing – original draft:** Guang Yang, Haoqiang He, Hongzheng Li.

**Writing – review & editing:** Guang Yang, Haoqiang He, Hongzheng Li.
